# Atypical Squamous Cells of Undetermined Significance: Bethesda Classification and Association with Human Papillomavirus

**DOI:** 10.1155/2011/904674

**Published:** 2011-06-29

**Authors:** Ana Cristina Macêdo Barcelos, Márcia Antoniazi Michelin, Sheila Jorge Adad, Eddie Fernando Candido Murta

**Affiliations:** ^1^Discipline of Gynecology and Obstetrics, Oncology Research Institute (IPON), UFTM, 38025-440 Uberaba, MG, Brazil; ^2^Discipline of Special Pathology, UFTM, 38025-440 Uberaba, MG, Brazil; ^3^Discipline of Immunology, Oncology Research Institute (IPON), UFTM, 38025-440 Uberaba, MG, Brazil

## Abstract

*Introduction*. To analyze patients with atypical squamous cells of undetermined significance (ASCUS) through a cytology review and the presence of microbiological agents, with consideration of colposcopy and semiannual tracking. *Methods*. 103 women with ASCUS were reviewed and reclassified: normal/inflammatory, ASCUS, low-grade squamous intraepithelial lesion (LSIL), or high-grade squamous intraepithelial lesion (HSIL). If ASCUS confirmed, it was subclassified in reactive or neoplastic ASCUS, ASC-US, or ASC-H; and Regione Emilia Romagna Screening Protocol. Patients underwent a colposcopic examination, and test for *Candida* sp., bacterial vaginosis, *Trichomonas vaginalis*, and human papillomavirus (HPV) were performed. *Results*. Upon review, ASCUS was diagnosis in 70/103 (67.9%), being 38 (54.2%) reactive ASCUS and 32 (45.71%) neoplastic ASCUS; 62 (88.5%) ASC-US and 8 (11.41%) ASC-H. ASCUS (Regione Protocol), respectively 1-5: 15 (21.4%), 19 (27.1%), 3 (27.1%), 16 (22.8%), and 1 (1.4%). A higher number of cases of cervical intraepithelial neoplasia (CIN) II/III in the biopsies of patients with ASC-H compared to ASC-US (*P* = .0021). High-risk HPV test and presence of CIN II/III are more frequent in ASC-H than ASC-US (*P* = .031). *Conclusions*. ASC-H is associated with clinically significant disease. High-risk HPV-positive status in the triage for colposcopy of patients with ASC-US is associated with increased of CIN.

## 1. Introduction

Since Papanicolaou's introduction of the Pap smear (1943), a variety of terms have been used to describe accompanying cytological diagnoses [1]. The Bethesda classification proposed in 1988 (revised in 1991) reflects the biological behavior of squamous intraepithelial lesions (SILS) of the cervix [2]. This classification scheme subdivides abnormal squamous epithelial cells into four groups: (1) atypical squamous cells of undermined significance (ASCUS); (2) low-grade squamous intraepithelial lesions (LSILS), encompassing light dysplasia/cervical intraepithelial neoplasia (CIN) I as well as cell changes associated with the human papillomavirus (HPV); (3) high-grade squamous intraepithelial lesions (HSIL), including moderate dysplasia/CIN II, severe dysplasia, and carcinoma *in situ*/CIN III; (4) squamous cell carcinoma.

The ASCUS diagnosis has caused confusion and controversy with respect to its significance and appropriate use [3–5]. More than 2 million women in the United States receive a diagnosis of ASCUS in cervical-vaginal cytology each year [4–6]. The ideal clinical tracking of these women has been highly controversial, and doubts remain regarding which control and treatment strategies are best [3, 7].

Comparisons between laboratories have shown that the diagnostic frequency of ASCUS varies from 1.6% to 9% [8, 9]. Gerber et al. detected ASCUS in 5% (1,387) out of a total of 29,827 patients in their study [9]. Lee et al. reported 1,035 ASCUS diagnoses out of 49,882 oncological cytologies performed, a 2% frequency rate [10]. According to some authors, the frequency of ASCUS should not exceed 2- to -3 times the frequency of LSIL [2–11]. 

Guerrini et al. have attempted to better define the ASCUS diagnosis by using the morphological criteria recommended by the Regione Emilia Romagna Screening Protocol (1997) [12]. This subdivision improved treatment courses, as cases of ASCUS at levels 1 to 3 corresponded to more substantial colposcopic findings which are related to the presence of CIN in biopsies [13]. 

The new Bethesda reclassification released in 2001 included a major change with respect to ASCUS. The category was subdivided into two categories: ASC-US (atypical squamous cells of undetermined significance), which reflects the difficulties in distinguishing between reactive changes and LSIL, and ASC-H (atypical squamous cells, cannot rule out a high-grade lesion), which reflects a differential diagnosis between immature reactive metaplasia and HSIL [10, 14]. Morin et al. showed the presence of CIN in 22.2% of the biopsies of 360 women with ASCUS, with 16.1% having CIN I and 5.3% having CIN II/III [1]. 

Research on HPV has begun to shape part of the evaluation of patients with ASCUS and other cytological changes [15]. The addition of a biomolecular test for HPV increases the sensitivity of detection for CIN in women with ASCUS relative to repeated cytology [16]. On the other hand, a negative test for HPV in women with ASCUS can reduce the number of times colposcopy is needed and reduce unnecessary biopsies [1, 17]. The HART (*HPV Testing in Addition to Routine Testing Study*), HPV testing with reflex results, was found to be highly sensitive for the diagnosis of subjacent CIN in women between the ages of 30 and 60, compared to a repetition of cytology [18]. 

The correct interpretation of the intensity of ASCUS morphological changes with respect to patient prognosis, the significance of the findings, and the appropriate clinical course to be followed have yet to be clarified. Therefore, the objectives of this study were (1) to evaluate the variation in the ASCUS diagnosis in routine Papanicolaou exams and other morphological classifications of ASCUS, (2) to analyze the microbiological agents that cause inflammation, and (3) to evaluate the use of HPV testing in the triage of patients with ASCUS based on colposcopy. 

## 2. Materials and Methods

### 2.1. Study Design

A prospective study was performed at the walk-in clinic of the Gynecology and Obstetrics Division of the Federal University of the Triângulo Mineiro between January 2003 and December 2007. In the first part of the study, women with a diagnosis of ASCUS were evaluated by cervical-vaginal cytology in a routine exam performed by four medical cytopathologists. Cytology findings were used in triage, and the order of patient entry followed the series sequence of the Pap smear registrations of the institution's cytopathology service. 

Patients with a diagnosis of ASCUS, who were taken to the colposcopy service, were informed about the study and its purpose. Those who agreed to participate signed terms of informed consent approved by the ethics committee of the Federal University of the Triângulo Mineiro. Women became part of the study when they met the inclusion criteria: a diagnosis of ASCUS by cytology; not pregnant; no bleeding during the exam; no use of oral antibiotics, fungicides, or vaginal creams in the last 30 days; sexual abstinence for at least two days; no previous history of SIL or cervical procedures.

### 2.2. Methods

#### 2.2.1. Clinical Methods

Information about the age, habits, and lifestyles (parity, number of partners, age of sexarche, age of first pregnancy, and smoking status), contraceptive methods used, and history of sexually transmitted diseases was initially collected.

#### 2.2.2. Microbiological and Biochemical Methods

After providing the above data, the patients underwent a gynecological examination, colposcopy, and collection of cervical-vaginal material for the study of microbiological agents and HPV by the hybrid capture technique. A variety of procedures were used to collect the samples as described hereafter. To search for *Trichomonas vaginalis*, material was collected from the base of the vaginal fornix with a swab, and the secretion was placed on a glass slide. After adding drops of saline solution and placing a coverslip on the specimen, a search for moving and flagellated microorganisms was performed by optical microscopy (fresh examination). To search for *Candida sp.*, vaginal material was collected with a swab and seeding performed in a Petri dish, containing the Sabouraud Agar culture medium, where the growth of fungus was verified. To search for bacterial vaginosis, the widely accepted clinical criteria originally proposed by Amsel et al. were used [19]: (1) homogenous vaginal secretion that adheres to the vaginal walls, (2) vaginal pH above 4.5, (3) the presence of a characteristic smell after adding a solution of 10% potassium hydroxide to the vaginal secretion, and (4) the presence of “clue cells” in the Gram-colored smear. The presence of 3 out of 4 of the above criteria was considered sufficient for establishing a diagnosis. Measurement of pH was performed by collecting vaginal material using a swab and then introducing it into a sterile test tube, containing 1 mL of distilled, deionized water. The material was taken to the laboratory at the end of the consult, and vaginal pH was gauged using a designated digital Sentron brand pH meter, which uses a 0 to 14 pH scale [20]. For this determination, the contents of the test tube were used. After homogenization in a vortex for about 10 seconds, the swab was removed and introduced into the flask of the pH measuring machine, with an electrode located at its end. Quantification of pH was done digitally on the spot. This procedure took place no longer than 1 hour after each sample had been collected. To search for human HPV, endocervical and ectocervical material was initially collected with a special brush, a component of the Digene hybrid capture kit, and placed in its own tube containing material to preserve it, and then maintained frozen at a temperature of −20°C. At the end of the collection period, once a sufficient number of samples had been obtained, the tubes were defrosted and analyzed according to hybrid capture techniques.

**Hybrid Capture**

The Hybrid Capture II *System *DML 2000 brand microplate system machine with signal amplification was used for chemiluminescence. The information and methodology described below were derived from the instruction manual provided by the product vendor (Digene of Brazil) and are consistent with previously described techniques [20].

The *kit *used to detect HPV had 18 viral types grouped into two pools of probes. The probes for low-risk virus included types 6, 11, 42, 43, and 44, representing approximately 70% of this viral group. With respect to the high-risk virus, the system had probes for types 16, 18, 31, 33, 35, 39, 45, 51, 52, 56, 58, 59, and 68, representing approximately 99% of this viral group. According to the vendor, the microplating sensitivity for HPV is 1 pg/mL, equivalent to 0.1 copies of the virus.

**Colposcopic Exam**

After the materials had been collected, the patients underwent video-colposcopy with image capture. The Barcelona classification scheme [21], proposed in 2002, was used to describe the findings. Briefly, samples were divided into two classes: normal colposcopic findings (original squamous epithelium, columnar epithelium, and normal transformation zone) and abnormal colposcopic findings (acetowhite epithelium, spotted, mosaic, leucoplasia, iodine-negative zone, and atypical vessels), with the latter category subdivided into minor or major changes depending on the intensity of the changes observed. The following reagents were used to perform the colposcopy: aqueous solution of 3% glacial acetic acid, lugol's solution, and sodium bisulfite. When changes were observed, the patients subsequently underwent a directed biopsy using Gaylor-Medina forceps.

**Cytological Evaluation and Histopathology**

In a second step, the smears of patients with an initial diagnosis of ASCUS by the cytopathologists during the routine exam were reviewed and reclassified by the same cytopathologists, in collaboration with the author of this study, as normal/inflammatory cytology, ASCUS, LSIL, or HSIL. When a diagnosis of ASCUS was confirmed upon review, the case was subclassified as either probably reactive or probably neoplastic (Bethesda 1988–1991) and, also according to the Bethesda 2001 norms, as ASC-US or ASC-H. Cases of ASCUS were newly subclassified according to their morphological changes following the recommendations of the Regione Emilia Romagna Screening Protocol [12] into the following groups: atypical squamous cells with mature-intermediate type cytoplasm (ASCUS 1), metaplastic atypical squamous cells (ASCUS 2), atypical squamous cells with parakeratosis (ASCUS 3), reactive atypical cells (ASCUS 4), and atypical squamous cells with atrophy (ASCUS 5).

The cytology smear was colored using the Papanicolaou technique and evaluated according to the morphological criteria of amphophilia, perinuclear halo, dyskeratosis, nuclear criteria (binucleation, multinucleation), increase in the nucleus/cytoplasm relation, anisokaryosis, hyperchromasia, nuclear atypias, and karyorrhexis. The biopsies were also reviewed and reclassified by the same pathologist, in conjunction with the author of this study. The patients underwent new cytological and colposcopic evaluations after 6 months. The results were analyzed by comparing cytological and colposcopic criteria, the presence of microbiological agents that cause infection and HPV in patients with ASCUS of a probably reactive character and ASCUS of a probably dysplastic character, ASC-US, and ASC-H.

#### 2.2.3. Statistical Analysis

The GraphPad InStat program, version 3.0, was used for statistical analysis. The results were compared using Fisher's exact test with a significance level of less than 5% (*P* < .05).

## 3. Results

Between January 1, 2003, and December 31, 2007, 46,362 Pap smears were performed by the cytopathology service of the Gynecology and Obstetrics Division. Of these, 41,349 (89.18%) had a negative cytology for oncological changes, 2,309 (4.98%) had a diagnosis of ASCUS, 265 (0.57%) had a diagnosis of AGUS (atypical glandular changes of an undermined significance), 1,760 (3.79%) had a diagnosis of LSIL, 551 (1.18%) of CIN II/III, and 128 (0.27%) of invasive carcinoma of the cervix. The screening is opportunistic and covers some areas of Uberaba (Minas Gerais, Brazil) and some neighboring towns. The population served is of low socioeconomic status.

A total of 103 women with an initial diagnosis of ASCUS were randomly selected and evaluated. Their average age was 35.76 (range, 18–50 years old). Of the 103 participants, 32 (31%) were smokers. In terms of contraceptive use, 40 (38.83%) had received tubal ligation, 30 (29.12%) used hormonal methods, 3 (2.91%) used only condoms, 1 (0.97%) used an intrauterine device, and 29 (28.15%) did not use any form of contraceptive. The women's average number of sexual partners was 2.15 (range, 1–10). The average age of their first sexual relationships was 17.62 years old (range, 12–27 years old) and the average age of their first pregnancy was 19.67 years old (range, 12–31 years old). Sixteen women (15.53%) were nulliparous, 60 (58.25%) had between one and three children, and 27 (26.21%) had more than three children.

The slides of all 103 of these initial cases of ASCUS were reviewed by the same examiner in conjunction with the author of this study, evaluating a variety of changes according to the protocol. Of the 103 cases, 70 (67.96%) were confirmed as ASCUS at the second reading; 30 (29.12%) were reclassified as normal/inflammatory smears, 2 (1.94%) as LSIL, and 1 (0.97%) as HSIL. Of the 70 cases of ASCUS, 38 (54.28%) were reclassified as ASCUS of a probably reactive nature and 32 (45.71%) as ASCUS of a probable neoplastic nature (Bethesda, 1991); 62 (88.57%) were reclassified as ASC-US and 8 (11.41%) as ASC-H (Bethesda, 2001). Patients with a diagnosis of SIL at the review were excluded from the reporting of the results.

The results of the microbiological examinations for *Candida sp.*, bacterial vaginosis. and *T. vaginalis* of 100 of the patients reevaluated in this study are shown in Table 1. No statistically significant difference was found in the comparison of the presence of infection (bacterial vaginosis, *Candida sp.* and *T. vaginalis*) between the group with probable reactive ASCUS and probable neoplastic ASCUS. A statistically significant difference was observed, however, with respect to the presence of infection between the ASCU-US and ASC-H groups.

The colposcopic findings are shown in Table 2. The colposcopy was considered unsatisfactory when it was not possible to see the squamocolumnar junction. When comparing the presence of abnormal colposcopic findings in the probably reactive and probably neoplastic groups, a statistically significant difference was found, with more abnormal findings being observed in the neoplastic group. When the same groups were compared, but only major colposcopic findings were evaluated, the difference remained significant, being greater in the group with changes that were probably neoplastic. Analysis of the colposcopic findings in the patients with cytology reviewed according to the Bethesda 2001 classification revealed significantly more abnormal colposcopic findings in the ASC-H group than in the ASC-US group. A similar comparison for major colposcopic findings also revealed significantly more incidences in the ASC-H group relative to the ASC-US group.

Among the 70 patients with ASCUS after cytology review, 30 (42.8%) underwent biopsy. Five of these (16.6%) were infected with HPV, 9 (30%) had CIN (3 CIN I, 3 CIN II and 3 CIN III), and 16 (53.3%) had normal biopsies. HPV infection was present in 4 patients with probable neoplastic ASCUS and in 1 patient with probable reactive ASCUS; in the Bethesda 2001 classification, the 5 HPV cases all involved patients with ASC-US that were subjected to biopsies. Three cases of CIN I were present in biopsies of patients with probable reactive ASCUS (Bethesda 1991) and ASC-US (Bethesda 2001). CIN II was present in 1 patient with reactive ASCUS and 2 with probable neoplastic ASCUS; and in the 2001 classification, all cases of CIN II were present in the biopsies of patients with ASC-H. All three CIN III diagnoses were made in patients in the neoplastic ASCUS and ASC-H groups. In the group of 30 patients with normal/inflammatory cytology, 8 underwent biopsy, revealing CIN I in 1 case and HPV infection in 1 case. 

The anatomopathological results of biopsies performed in patients with changes found during the colposcopic examination are shown in Table 3. No statistically significant differences were found when the presence of changes was compared between the normal/inflammatory, probably reactive ASCUS, and probably neoplastic ASCUS groups. The reconfirmed cases of ASCUS (*n* = 70) were then subclassified according to the recommendations of the Regione Emilia Romagna Screening Protocol as follows: 15 cases of ASCUS 1 (21.4%), 19 of ASCUS 2 (27.1%), 19 of ASCUS 3 (27.1%), 16 of ASCUS 4 (22.8%), and 1 case of ASCUS 5 (1.42%). The results of the biopsies showed 3 cases of CIN II/III among patients with ASCUS 1, 2 cases among patients with ASCUS 2, and 1 case among patients with ASCUS 4. There were no cases of CIN II/III among the patients with ASCUS 3 and 5. When the results of the colposcopic findings as classified according to the Regione Emilia Romagna were analyzed, there were no statistically significant group differences.

When the results of the biopsies were reevaluated against the cytologies according to Bethesda 2001 (Table 4), we found a greater frequency of CIN II/III in the group of patients with ASC-H, compared to the group with normal/inflammatory cytology. There were a greater number of cases of CIN II/III in the biopsies of patients with ASC-H relative to the group with ASC-US. When CIN II/III findings were compared between the morphological ASCUS groups, we found a significantly greater frequency among patients with ASC-H than those with ASCUS 1 and those with probable neoplastic ASCUS. The distribution of cases of CIN II/III (absent/present – *n*, %) at biopsy in relation to the cytological diagnoses of probably neoplastic ASCUS, ASC-H, and ASCUS 1 (Regione Emilia Romagna Classification) showed 27/5 (84.3%/15.6), 12/5 (80/20), and 3/5 (37.5/62.5), respectively, Bethesda 1991, probably neoplastic ASCUS, Regione Emilia Romagna, ASCUS 1 and Bethesda 2001, ASC-H, being *P* = .0713 and *P* = .0145, respectively, Bethesda 2001, ASC-H versus ASCUS 1 and probably neoplastic ASCUS (Fisher's exact test).

 With regard to the hybrid capture test in the 100 cases with an initial diagnosis of ASCUS, the presence of high-risk HPV DNA was detected in 19 (27.1%) women with reconfirmed ASCUS and in 8 (26.6%) with normal cytology on review. There was a greater frequency of high-risk HPV infection in the ASC-H group compared to the ASC-US group. The results of DNA/high-risk HPV experiment (negative/high-risk HPV – *n*, %) using the hybrid capture technique in patients with an initial diagnosis of ASCUS who, upon review, were reclassified as having cytologies that were normal/inflammatory, ASC-US and ASC-H showed 22/8 (73.3/26.6), 48/14 (77.4/22.6), 3/5 (37.5/62.5), respectively, normal, ASC-US, and ASC-H cytologies, being *P* = .0296, ASC-H versus ASC-US for high-risk HPV (Fisher's exact test). Of the 6 cases of high-grade CIN, after a biopsy guided by colposcopy (out of the 100 cases evaluated in the study), 5 (83.3%) tested positive for high-risk HPV by the hybrid capture technique.  Table 5 shows the correlations between ASC-US, ASC-H, and normal/inflammatory biopsies and the presence of high-risk HPV and the biopsy of high-grade lesions. We found a greater proportion of CIN II/III in the biopsies of patients testing positive for high-risk HPV in the ASC-H group than in the ASC-US group.

Out of a total of 70 patients with a diagnosis of ASCUS, after review, 6 were transferred to the oncology service for treatment because of biopsies showing CIN II or III. Of the 64 remaining cases, 7 did not come for followups. Of the 57 patients diagnosed with ASCUS, 43 had normal/inflammatory cytology and normal colposcopy at their semi-annual followup, 7 had ASC-US and normal colposcopy, and 7 had LSIL (4 CIN I and 3 HPV; with all cases of CIN I being proven by directed biopsy). Of these, all cases of CIN I and II were diagnosed in the first biopsy. Of the 4 cases of CIN I, 3 came from the initial group with probable neoplastic ASCUS and 1 came from the probably reactive ASCUS group. Based on the 2001 classification, all CIN I cases belonged to the ASC-US group. Of the 3 patients with HPV, 1 came from the initial probably neoplastic ASCUS group and 2 came from the probably reactive group; all 3 belonged to the ASC-US group.

Of the 30 patients for whom the diagnosis of ASCUS was not confirmed upon cytological reclassification, no tracking was performed. At their semi-annual followups, 25 (83.3%) had normal colposcopic exams and normal/inflammatory cytology and 3 had ASC-US. There were no cases of CIN in semesterly tracking in patients who had a normal/inflammatory cytology diagnosis on reclassification.

## 4. Discussion

A diagnosis of ASCUS not only depends on well-defined cytological patterns, but also on many subjective criteria [6, 12]. The reproduction of the interpretation of ASCUS is lower than 50% [6]. We observed that the number of cytological diagnoses of ASCUS in our service was equivalent to 1.3× the frequency of LSIS, or 4.98% of all cytologies performed. ASCUS frequency rates in the literature vary from 2% to 7% [10, 22, 23]. The present results from our service demonstrate an ASCUS frequency compatible with the Bethesda expectations.

In the women with a confirmed diagnosis of ASCUS, altered biopsies were found in 20% of the cases, with 11.4% of these being HPV/CIN I and 8.6% being CIN II/III. Some studies have reported a concomitance of CIN II/III in 5–10% of patients with ASCUS [1], results similar to those from our study. Roche and Spicer, who tracked patients with ASCUS for 2 years, reported 18% of cases with HPV/CIN I and 15% with CIN II/III [24]. Eltabbakh et al. found a 15.9% frequency of neoplasia in patients with ASCUS [25]. A prior study performed by our group evaluating 1,244 women with ASCUS revealed CIN I in 60.3%, CIN II/III in 17.46%, and invasive neoplasia in 6.3% of the cases [26]. Thus it was concluded that CIN or invasive lesions can occur in women with ASCUS, and therefore new cytology or colposcopy and rigorous tracking should be considered for these patients.

All patients in our study with a diagnosis of ASCUS, upon review, were reclassified as having lesions of a probable neoplastic nature (45.7%) or of a probable reactive nature (54.2%), a result quite similar to previous work by our group (45.4% probable neoplastic ASCUS and 54.5% probable reactive ASCUS after reviewing the ASCUS cytologies) [13]. Other studies in the literature have also made this subdivision. Guerrini et al. reclassified 107 patients with a diagnosis of ASCUS and found 78.5% of them to be probably reactive and 21.5% to be probably neoplastic [12]. The absence of well-defined criteria, as well as the subjectivity of the diagnosis, may account for this variation. After reclassification according to the Regione Emilia Romagna, our findings showed the presence of CIN II/III in 20% of the ASCUS 1 cases, 10.5% of the ASCUS 2 cases, 1% of the ASCUS 4 cases, and none of the ASCUS 3 and 5 cases. We observed a higher frequency of CIN II/III in the biopsies of patients with ASCUS 1, a finding similar to that of Guerrini et al. [12].

Women with a cytological diagnosis of ASC-H have been shown to have a higher association with CIN II/III than those with ASC-US [14]. Barreth et al. studied 517 women with a cytology of ASC-H, and found a 2.9% presence of cervical cancer, 1.7% with *in situ* adenocarcinoma, and 65.6% with CIN II/III [27]. In another study, 85 women with ASC-H underwent colposcopies and histological analysis of biopsies of the areas of change, with CIN II and III being found in 52 (61.2%) of the cases and invasive cancer being found in 7 (8.2%) of the cases [28]. Similar results were found in our study, which verified levels of 62.6% of CIN II/III for patients classified as ASC-H. The diagnosis of ASC-H in the Papanicolaou exam is associated with the risk of clinically significant disease, and a biopsy directed by colposcopy should be considered the proper course of action in these cases. 

 We performed a comparison of the Bethesda 1991, Bethesda 2001, and Regione Emilia Romagna classifications to better diagnose CIN II/III. We did not find any prior studies in the literature that had compared these three classification systems. When we performed the analysis, we observed a higher frequency of CIN II/III in cases of ASC-H than in cases of probable neoplastic ASCUS and ASCUS 1. Nevertheless, a limitation of our study is a low number of CIN II/III cases. 

The detection of high-risk HPV DNA is thought to be useful in supplementing an abnormal cytological result [29, 30] and that in women with ASCUS, the presence of an HPV-positive group can substantially increase the chances of finding CIN II/III and cervical cancer, even though in the majority of these women significant lesions are not found [6]. Therefore, some authors recommend that testing for HPV DNA be performed on women with ASCUS [15, 31], while a biopsy guided by colposcopy is only recommended if high-risk HPV is present, while other women are treated more conservatively [5].

Various studies have shown a difference in the presence of HPV DNA when the cytologies of ASC-US and ASC-H are compared. Srodon et al. studied patients with ASCUS classified according to the Bethesda 2001 system and found HPV to be present in 67% of women with ASC-H and 45% of women with ASC-US [32]. In evaluating the presence of high-risk HPV by hybrid capture, Kurman and Solomon found it to be present in 14.2% of ASC-US cases and 66.6% of ASC-H cases [2]. In our study, we found high-risk HPV in 22.5% of patients with ASC-US and 62.5% of patients with ASC-H. When the cytologies of ASC-US and ASC-H were compared, the presence of high-risk HPV DNA was statistically greater in the latter, highlighting the importance of this division in the detection of clinically significant disease.

Srodon et al. evaluated the presence of HPV and high-grade CIN in patients with ASC-US and ASC-H and found CIN II/III in 10.2% of HPV-positive patients with ASC-US and in 5.9% of HPV-negative patients with ASC-US [32]. We found that 7.14% of the cases of high-grade CIN II/III were in HPV-positive women with ASC-US. Furthermore, we found higher rates of CIN II/III in HPV-positive patients with ASC-US than in HPV-negative ASC-US group (no cases of high-grade CIN). The presence of CIN II/III in 7.14% of the HPV-positive women with ASC-US suggests that the HPV test could be used in the triage of patients with ASC-US for a colposcopy and that HPV positivity may be associated with an increased probability of CIN. The diagnosis of ASC-H appears to be associated with an increased risk of clinically significant lesions, especially when associated with oncogenic HPV. Excision of the lesion may be indicated because the risk for histologic CIN II found was high for women with HPV positive tests, HSIL cytology, and a high-grade impression at colposcopy [33]. Other methods as liquid-based cervical cytology do not show more efficacies in diagnosis HSIL compared to Pap smears [34]. Actually, the frontiers of cervical cancer prevention is the preventing persistent HPV infections using vaccination or HPV testing utilizing opportunistic or organized screening [35].

We believe that the data obtained in this study provide an important confirmation of the usefulness of both the ASCUS subdivision according to the Bethesda 2001 protocol and the hybrid capture test. We propose that an initial cytology of ASC-US be reviewed for diagnostic confirmation and that, if it stands, two strategies should be followed: (1) an HPV test should be performed, and (2) if there is a positive high-risk HPV finding, there should be a colposcopy or semi-annual followup with cytology. In cases classified as normal after a review of the ASC-US diagnosis, we suggest a new cytology be performed after 6 months. This finding is in agreement with the data obtained by Chen et al. [36], whose findings also lead to the conclusion that a cytology of ASC-US, especially without a prior Pap smear, is quite likely to develop into cervical cancer and that of the most aggressive kind. Another study about ASC-H showed that HPV DNA testing has an extremely high negative predictive value for histologic CIN II/III, reaching 100% in women 40 years and older [37]. Based on 2006 consensus guidelines, a program of DNA testing for high-risk types of HPV, repeat cervical cytologic testing, or colposcopy are all acceptable methods for managing women over the age of 20 years with ASC-US [38]. 

This study allows us to conclude that the level of agreement between observers in diagnosing ASCUS upon review was 67.96%. The frequency of infection was higher in patients with ASC-US than in patients in the ASC-H group, and the frequency of abnormal colposcopic findings was greater in the neoplastic ASCUS and ASC-H groups than in the reactive ASCUS and ASC-US groups, respectively. A positive HPV test, used in the triage of patients with ASC-US for colposcopy indication, appears to be associated with an increased chance of detecting CIN.

## Figures and Tables

**Table 1:** Distribution of patients with an initial diagnosis of ASCUS which, upon review, were reclassified as normal/inflammatory, ASCUS of a probably reactive nature, and ASCUS of a probably neoplastic nature, in relation to infection.

	Cytology				
Agent	Normal/inflammatory *N* (%)	ASCUS			
		Probably reactive *N* (%)	Probably neoplastic *N* (%)	ASC-US *N* (%)	ASC-H *N* (%)
*Candida sp.*	4 (13.4)	9 (23.6)	4 (12.5)	13 (21)	0
Bacterial vaginosis	2 (6.6)	6 (15.8)	6 (18.7)	12 (19.3)	0
*Trichomonas vaginalis*	0	2 (5.2)	0	2 (3.2)	0
No infection	24 (80.0)	21 (55.2)	22 (68.7)	35 (56.4)	8 (100)

Total cases by group	30	38	32	62*	8

*P = .0196 versus ASC-H, Fisher's exact test.

**Table 2:** Principal colposcopic findings in patients with an initial diagnosis of ASCUS who, upon review, were reclassified as normal/inflammatory cytology, probably reactive ASCUS, probably neoplastic ASCUS, ASC-US, and ASC-H.

	Cytology				
Colposcopy	Normal/inflammatory *N* (%)	ASCUS			
		Probably reactive *N* (%)	Probably neoplastic *N* (%)	ASC-US *N* (%)	ASC-H *N* (%)
Normal findings	17 (56.6)	23 (60.5)	9 (28.1)	32 (51.6)	0
Abnormal findings Major*	0	0	6 (18.7)*	1 (1.6)	5 (62.5)**
Minor	8 (26.6)	9 (23.6)	15 (46.8)	21 (33.9)	3 (37.5)
Unsatisfactory	5 (16.6)	6 (15.8)	2 (6.2)	8 (12.9)	0

Total cases by group	30	38	32	62	8

*P = .0020 and *P* = .0018 for the presence of abnormal and major abnormal colposcopic findings, respectively, versus the probably reactive ASCUS group, Fisher's exact test.

**P = .0017 and *P* < .0001 for the presence of abnormal and major abnormal colposcopic findings, respectively, versus the ASC-US group, Fisher's exact test.

**Table 3:** Results of biopsies performed on patients with an initial diagnosis of ASCUS who, upon review, were reclassified as having normal/inflammatory cytology, probably reactive ASCUS, and probably neoplastic ASCUS, and who, in accordance with the Regione Emilia Romagna (ASCUS 1 to 5), showed evidence of changes in the colposcopic exam.

	Cytology							
Biopsy	Normal/inflammatory *N* (%)	ASCUS						
		Probably reactive *N* (%)	Probably neoplastic *N* (%)	1 *N* (%)	2 *N* (%)	3 *N* (%)	4 *N* (%)	5 *N* (%)
Normal	6 (20.0)	7 (18.4)	9 (28.1)	1 (6.6)	5 (26.3)	5 (26.3)	4 (25.0)	1 (100.0)
HPV/CINI	2 (6.6)	1 (2.6)	7 (21.8)	2 (13.3)	4 (21.0)	2 (10.5)	0	0
CIN II/III	0	1 (2.6)	5 (15.6)	3 (20.0)	2 (10.5)	0	1 (6.25)	0
Not performed	22 (73.3)	29 (76.3)	11 (34.3)	9 (60.0)	8 (42.1)	12 (63.1)	11 (68.7)	0

Total cases by group	30	38	32	15	19	19	16	1

All *P*s > .05; Fisher's exact test.

**Table 4:** Results of the biopsies performed on the total group of patients with an initial diagnosis of ASCUS who, after review, were reclassified as having cytologies that were normal/inflammatory, ASC-US, and ASC-H, which showed changes in the colposcopic examination.

	Cytology		
Biopsy	Normal/inflammatory *N* (%)	ASCUS	
		ASC-US *N* (%)	ASC-H *N* (%)
Normal	6 (20.0)	13 (21.0)	3 (37.5)
HPV/CIN I	2 (6.6)	8 (13.0)	0
CIN II/III	0	1 (1.6)	5 (62.5)*
Not performed	22 (73.3)	40 (64.5)	0

Total cases by group	30	62	8

*P = .0256 and *P* = .0021, relative to ASC-US group, versus ASC-H group with normal/inflammatory cytology and cases of CIN II/III in biopsies of patients with ASC-H, respectively, Fisher's exact test.

**Table 5:** Presence of CIN II/III in biopsies, in relation to positivity for high-risk HPV through hybrid capture, and reclassification of ASCUS cytology according to Bethesda 2001.

Cytology	High-risk HPV positive			High-risk HPV negative		
	Biopsy with CIN II/III *N* (%)	Biopsy without CIN II/III *N* (%)	Biopsy not performed *N* (%)	Biopsy with CIN II/III *N* (%)	Biopsy without CIN II/III *N* (%)	Biopsy not performed *N* (%)
ASC-US	1 (20.0)	7 (70.0)	6 (50.0)	0	14 (63.6)	34 (68.0)
ASC-H	4 (80.0)*	1 (10.0)	0	1 (100.0)	2 (9.0)	0
Normal	0	2 (20.0)	6 (50.0)	0	6 (27.3)	16 (32.0)

Total	5	10	12	1	22	50

*P = .0319, presence of CIN II/III at biopsy versus ASC-US among patients who tested positive for high-risk HPV, Fisher's exact test.
